# Robust C–C bonded porous networks with chemically designed functionalities for improved CO_2_ capture from flue gas

**DOI:** 10.3762/bjoc.12.220

**Published:** 2016-10-28

**Authors:** Damien Thirion, Joo S Lee, Ercan Özdemir, Cafer T Yavuz

**Affiliations:** 1 Graduate School of Energy, Environment, Water, Sustainability (EEWS), Korea Advanced Institute of Science and Technology (KAIST), Guseong Dong, Yuseong Gu, Daejeon 305–701, Korea; 2 Department of Chemistry, Korea Advanced Institute of Science and Technology (KAIST), Guseong Dong, Yuseong Gu, Daejeon 305–701, Korea

**Keywords:** C–C bond, CO_2_ capture, microporous materials, porous polymers, postmodification

## Abstract

Effective carbon dioxide (CO_2_) capture requires solid, porous sorbents with chemically and thermally stable frameworks. Herein, we report two new carbon–carbon bonded porous networks that were synthesized through metal-free Knoevenagel nitrile–aldol condensation, namely the covalent organic polymer, COP-156 and 157. COP-156, due to high specific surface area (650 m^2^/g) and easily interchangeable nitrile groups, was modified post-synthetically into free amine- or amidoxime-containing networks. The modified COP-156-amine showed fast and increased CO_2_ uptake under simulated moist flue gas conditions compared to the starting network and usual industrial CO_2_ solvents, reaching up to 7.8 wt % uptake at 40 °C.

## Findings

Porous polymers are network polymers that are made from multivalent monomers and form permanent pores through the net formation [1]. They differ slightly from cross-linked polymers with more certainty in the location of branching and the type and quantity of functionalities. Also, the rigidity of the monomers is essential since the porosity, a desired feature, is best achieved by structurally interlocked motifs. In industrial applications, porous polymers have to meet several important criteria like high porosity, chemical and thermal stability while also possessing application-specific functionalities [1–2]. Designing and synthesizing structures that respect all these criteria is a challenging task. Chemical and thermal stability can be achieved best by having robust C–C bonded architecture like in porous polymer networks (PPNs) [3], porous aromatic frameworks (PAFs) [4] or conjugated microporous polymers (CMPs) [5]. Most of these networks have the advantage of high porosity, but they are all made through precious metal-catalyzed reactions, which elevates the synthesis cost and limits their mass production. Another type of C–C bonded porous networks are hypercrosslinked polymers (HCPs) [6]. HCPs are mainly synthesized through Friedel–Crafts alkylation using iron chloride and thus, are not relying on precious metals. Unfortunately, Friedel–Crafts reactions are not very tolerant to functional groups like nitriles or amines [7]. On the other hand, structures incorporating heteroatoms, such as nitrogen in imine, Tröger’s base or azo linked networks [8–11], have led to materials with remarkable gas uptake properties. Compared to C–C bonded networks, C–O or C–N linked structures are less thermally stable or chemically resistant. Furthermore, the chemical reactions involved in the formation of these structures do not allow obtaining free nitrile or amine functionalities. The most used strategy to install available functional groups on porous materials is through post-modification, which often requires several steps and harsh conditions, and yields low porosity [12–13].

The Knoevenagel condensation of benzyl nitriles and aldehydes produces C–C bonded products with labile nitrile functionalities. In fact, nitrile groups have been shown in porous polymers (particularly in polymers of intrinsic microporosity (PIMs)) to be good precursors to several functionalities like carboxylic acids, tetrazoles, amines or amidoximes [14–17]. To the best of our knowledge the nitrile functionality has not been explored in other types of porous polymers, except our earlier work [18].

Herein we report the synthesis of two new cyanovinylene-bridged covalent organic polymers, indexed as COP-156 and COP-157, through Knoevenagel nitrile–aldol condensation. COP-156 showed a high surface area (650 m^2^/g) with a hierarchical porosity, allowing synthetic post-modifications. The nitrile functional groups were first reduced to amine groups (COP-156-amine) and then through reaction with hydroxylamine converted into amidoxime groups (COP-156-amidoxime). The COP-156-amine proved increased affinity towards CO_2_ with stronger binding energy, especially under moist conditions, reaching up to 7.8 wt % at 40 °C, a 2.1 wt % increase when compared to the original COP-156.

**Scheme 1:**
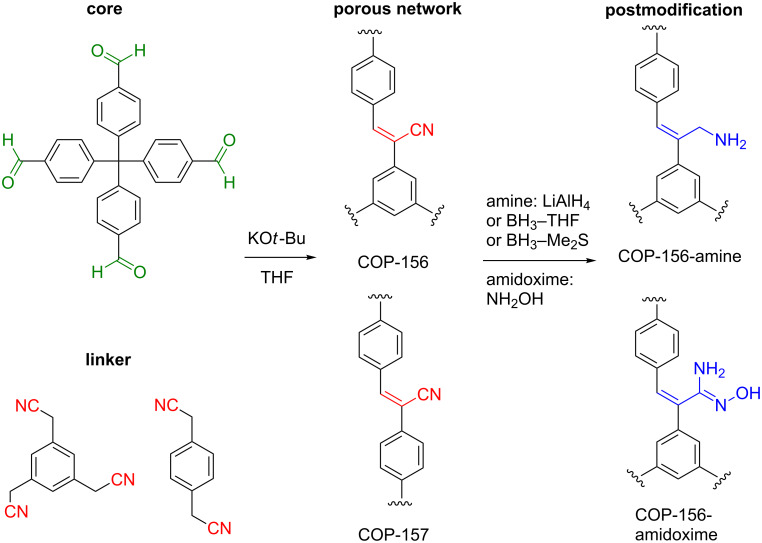
Synthesis route for COP-156 and COP-157, and the post-modification of COP-156.

Despite the promise, cyanovinylene bridged porous networks are not studied extensively and our previously reported COP-100 structure [18] suffered from low porosity. In order to obtain a cyanovinylene-bridged porous network with tangible surface area, different core and linker combinations were tested leading to the synthesis of COP-156 and COP-157 (Scheme 1). Tetraphenylmethane cores are well-known tetrahedral building blocks that are commonly employed to form highly porous materials [4,19]. A trivalent 1,3,5-phenylenetriacetonitrile linker led to COP-156 whereas a divalent 1,4-phenylenediacetonitrile formed COP-157 (Scheme 1). Despite having the same tetrahedral core, COP-156 showed a much higher surface area (650 m^2^/g) than COP-157 (146 m^2^/g). One possible reason is the framework interpenetration, where neighboring branches of a network polymer intertwine through the adjacent open pore. For these reasons, we studied COP-156 for post-modification.

COP-156 possesses labile nitrile functionalities, which can easily be transformed into amines or amidoximes. Amines are especially attractive for gas uptake [20] and amidoximes are shown effective in uranium capture [21]. COP-156 has been post-modified through two different routes, either by reduction to the corresponding amine or reaction with hydroxylamine into an amidoxime. For the reduction, three reagents (LiAlH_4_, BH_3_–THF and BH_3_–Me_2_S) were screened. All reductions were chemically successful, but the textural properties of the modified networks were noticeably different. BH_3_–Me_2_S gave the highest surface area and therefore CO_2_ uptake studies were based on this reduction method (see Supporting Information File 1, Table S1 for the properties of other obtained reduced networks). All networks were studied by FTIR, elemental analysis, TGA, and gas sorption experiments.

**Figure 1:**
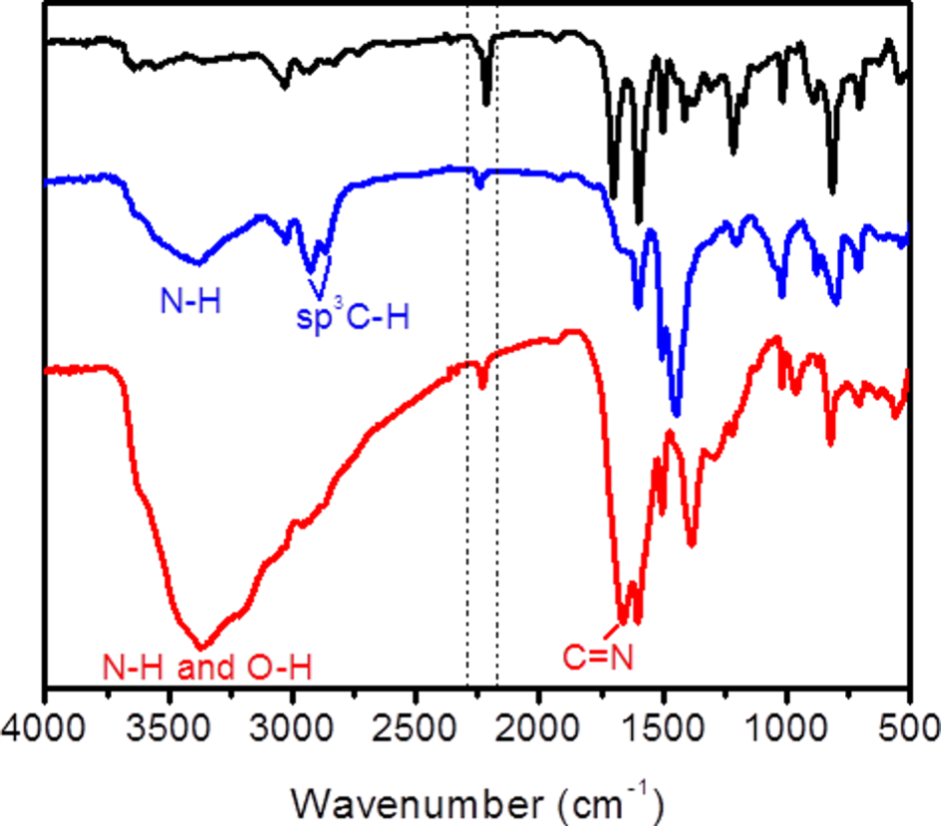
FTIR spectra of COP-156 (black), COP-156-amine (blue) and COP-156-amidoxime (red). The dotted lines highlight the characteristic stretching for the nitrile functionality (2215 cm^−1^), which loses intensity by the post-modification reactions.

The FTIR spectra of COP-156 shows the characteristic nitrile stretch at 2215 cm^−1^ and several bands for the carbon–carbon double bonds (C=C) of both aromatic and alkene between 1600 and 1350 cm^−1^ (Figure 1). The presence of a peak at 1698 cm^−1^ can be attributed to carbonyl units from unreacted aldehydes. This is further confirmed in elemental analysis showing the presence of 4–5 % oxygen (Supporting Information File 1, Table S2). The condensation reaction is very fast and the formed network precipitates in a matter of minutes, leading to unreacted aldehyde edges on the surface of the grains, explaining the leftover carbonyl units. COP-157 shows similar results due to the chemical similarity of the structures and the reaction conditions (Supporting Information File 1, Figure S1 and Table S2).

After post-modification, the nitrile stretching becomes very weak in both COP-156-amine and COP-156-amidoxime, confirming the chemical modification of the nitrile units (Figure 1). A broad region around 3400 cm^−1^ can be attributed to the NH stretching of the formed amine groups and the two peaks at 2931 and 2866 cm^−1^ belong to sp^3^ CH stretching of the formed methylene unit in COP-156-amine. For COP-156-amidoxime the 3400 cm^−1^ region is even broader, due to the combination of both NH and OH stretching. The strong peak at 1664 cm^−1^ can be attributed to the imine C=N bond of the amidoxime structure.

**Figure 2:**
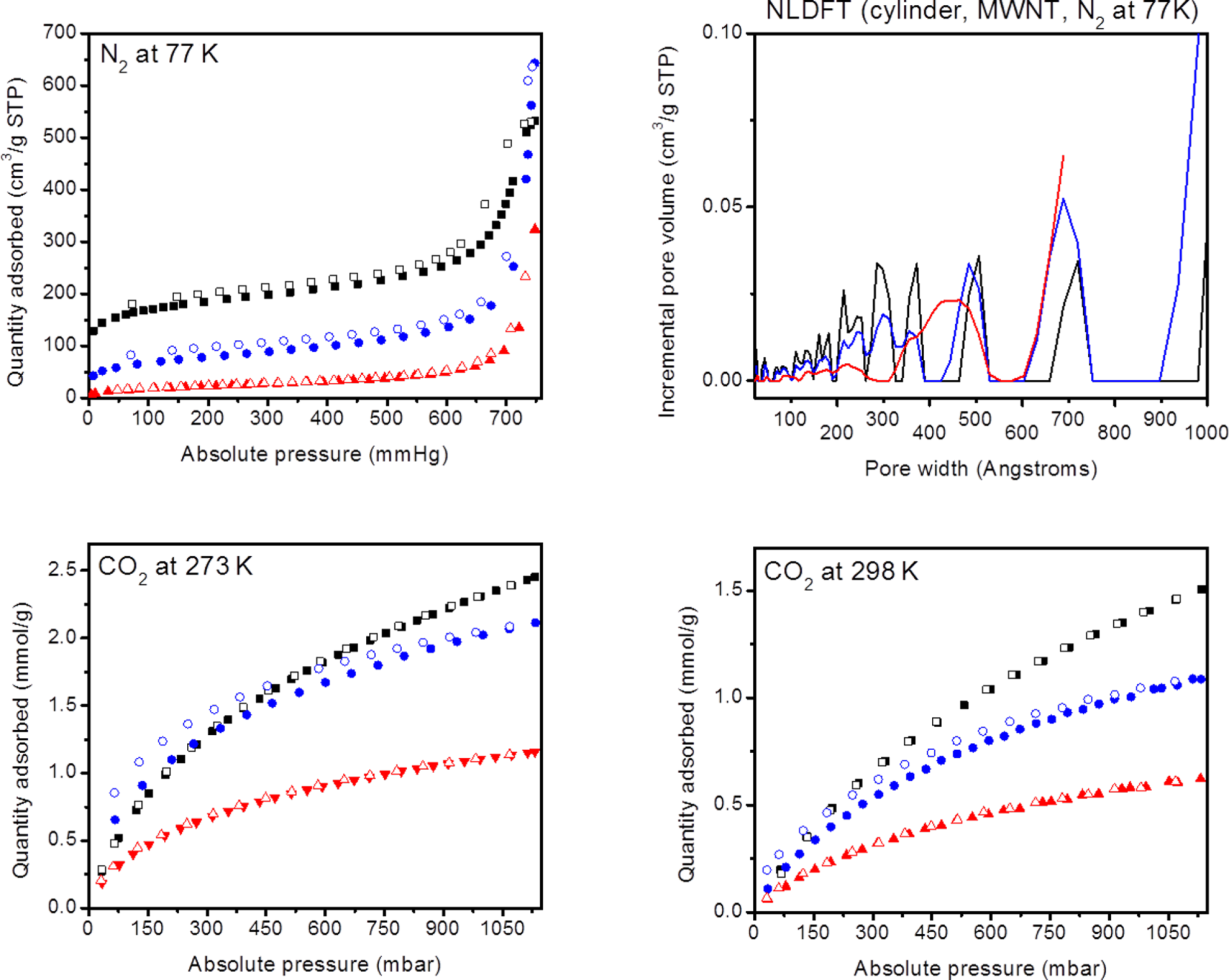
Gas adsorption (filled dots)-desorption (empty dots) isotherms and pore size distribution of COP-156 (black), COP-156-amine (blue) and COP-156-amidoxime (red).

The textural properties were measured through nitrogen sorption isotherms at 77 K and calculated based on the Brunauer–Emmett–Teller (BET) theory. COP-156 shows type II N_2_ sorption isotherms and a slight hysteresis, owing to the existence of micro, meso and macropores, with a surface area of 650 m^2^/g and a pore volume of 0.82 cm^3^/g at *P*/*P*_0_ = 0.99 (Figure 2). The pore size distribution is broad with several important pore sizes (2.2, 3.0, 5.0, 7.0 nm). Larger pores are required for post-modification, as the reagents may not be able to penetrate to the small micropores. CO_2_ sorption isotherms reflect the physisorptive nature of COP-156 with almost no hysteresis and an isosteric heat of adsorption (*Q*_st_) of 28.1 kJ mol^−1^ (Table 1, Figure 2 and Supporting Information File 1, Figure S3). COP-157 gave a similar isotherm but with lesser surface area (Supporting Information File 1, Figures S4 and S5). This could be explained by a higher interpenetration probability of the network structure than in COP-156, as the linker used for COP-157 is linear [22–23].

**Table 1:** BET surface area, CO_2_ sorption properties at 273 K, 298 K and heat of adsorption values of all networks.

Structure	BET surface area (m^2^/g)	CO_2_ at 273 K (mmol/g) 0.15/1 bar	CO_2_ at 298 K (mmol/g) 0.15/1 bar	*Q*_st_ (kJ/mol)^a^

COP-156	650	0.84/2.31	0.38/1.40	28.1
COP-157	146	0.45/1.18	0.22/0.71	29.5
COP-156-amine	263	0.94/2.00	0.34/1.09	49.9
COP-156-amidoxime	67	0.47/1.10	0.20/0.58	38.6

^a^Zero point coverage *Q*_st_ calculated from 273 K and 298 K.

After post-modification, the surface area diminishes, which is a common observation [13,24]. Yet, the pore volume of COP-156-amine increases to 0.99 cm^3^/g at *P*/*P*_0_ = 0.99, due to newly created pores from the reduction reaction (Figure 2). COP-156-amidoxime has decreased both surface area (67 m^2^/g) and pore volume (0.41 cm^3^/g at *P*/*P*_0_ = 0.99), as the functional group is larger than amine or nitrile. It should be noted that elemental analysis does not reach 100% with the measured elements (C, H, N, O), indicating the presence of trapped reagents in the pores. These could not be removed with extensive washing and therefore contribute to the porosity loss (Supporting Information File 1, Table S2). Nevertheless, CO_2_ sorption isotherms of COP-156-amine show a slight hysteresis, indicating that the network is not purely physisorptive anymore. At 0.15 bar (the relevant pressure of CO_2_ in flue gas emission) and 273 K, the uptake is slightly higher than the starting COP-156. The chemisorptive behavior and stronger binding affinity is reflected in the higher *Q*_st_ value of 49.9 kJ mol^−1^. The moderate binding energy is optimal for CO_2_ capture, as too strong binding requires high regeneration energy and raises the overall cost of the carbon capture operations [12,25].

Dry CO_2_ uptake is not always meaningful, especially with amine functionalities, as flue gas from power plants contains moisture [26]. When binding to CO_2_, amines go through either carbamate or carbamic acid formation. Carbamates form with the contribution of two amines and carbamic acid needs one amine and a water molecule. The presence of moisture, therefore, favors the formation of carbamic acids and leads to higher uptake capacity per sorbent mass. We performed moist CO_2_ uptake experiments in order to mimic an actual power plant flue gas on COP-156 and COP-156-amine (see Supporting Information File 1 for the experimental details).

**Figure 3:**
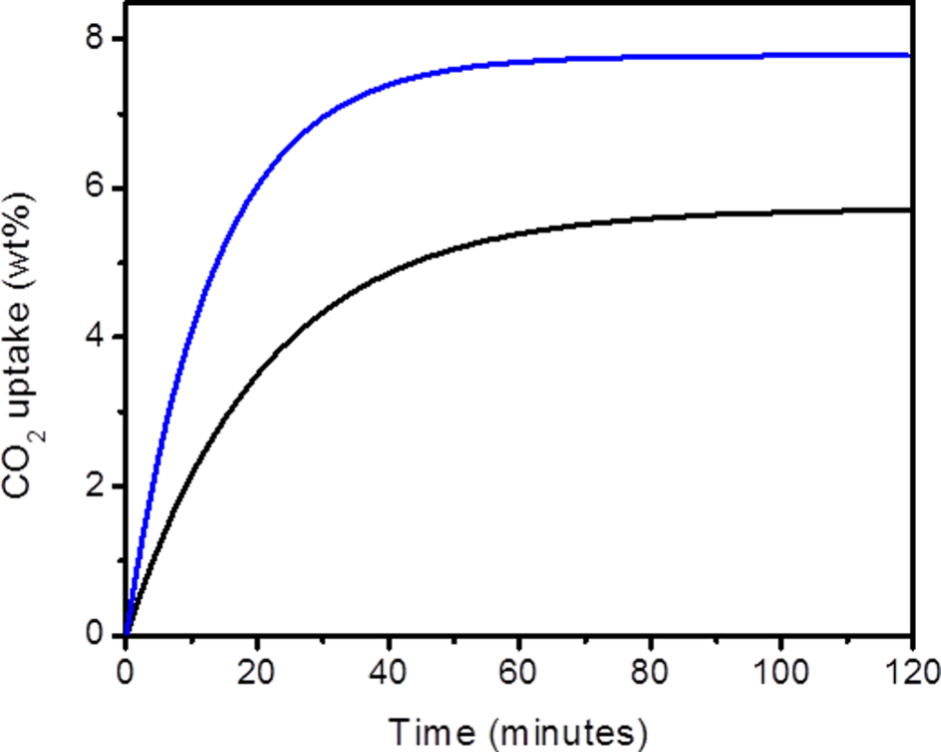
CO_2_ uptake under moist conditions at 40 °C of COP-156 (black) and COP-156-amine (blue).

As expected, COP-156-amine shows faster and higher uptake than COP-156 (Figure 3). For the COP-156-amine 80% of the total CO_2_ uptake is reached in 21 minutes, while COP-156 requires around 30 minutes to reach the same percentage. COP-156-amine yields a total uptake of 7.8 wt %, which is 2.1 wt % more than COP-156. These CO_2_ capacities are considerably higher than the industrial standard, monoethanolamine (MEA) (usually 2.1–5.5 wt %) [27–28].

In summary, we have shown that highly porous cyanovinylene networks could be obtained through Knoevenagel condensation of benzylic nitriles and aldehydes by choosing appropriate building blocks. The resulting nitrile functionalities in COP-156 could be easily modified into free amine or amidoxime groups. The amine functionalities in COP-156-amine allowed for higher CO_2_ binding strength and therefore led to fast and improved CO_2_ uptake in moist conditions.

## Supporting Information

File 1 Materials and methods, synthesis, FTIR spectra and gas sorption isotherms of COP-157, heat of adsorption graph, elemental analysis and TGA of all structures.
