# Supplementary material for: Robust C–C bonded porous networks with chemically designed functionalities for improved CO2 capture from flue gas
Source: Beilstein J Org Chem. 2016 Oct 28;12:2274–9. doi: 10.3762/bjoc.12.220 (PMC5238613; doi:10.3762/bjoc.12.220)
Supplement: File 1 — Materials and methods, synthesis, FTIR spectra and gas sorption isotherms of COP-157, heat of adsorption graph, elemental analysis and TGA of all structures. [file Beilstein_J_Org_Chem-12-2274-s001.pdf]

Supporting Information

for

Robust C–C bonded porous networks with chemically designed functionalities for improved CO_2 capture from flue gas

Damien Thirion[1], Joo. S. Lee[1], Ercan Özdemir[1] and Cafer T. Yavuz*[1,2]

Address. [1]Graduate School of Energy, Environment, Water, Sustainability (EEWS) and [2]Department of Chemistry, Korea Advanced Institute of Science and Technology (KAIST), Guseong Dong, Yuseong Gu, Daejeon 305–701, Korea, www.porouspolymers.com, +82-42-350-1718

Email: Cafer Tayyar Yavuz* - yavuz@kaist.ac.kr

*Corresponding author

Materials and methods

All chemicals and solvents were obtained from Sigma-Aldrich, Alfa Aesar, TCI Japan or Samchun Pure Chemicals, Korea and were used without further purification unless otherwise specified. THF was dried and collected from a Hansen Glass Contour Ultimate Solvent Purification 5 System. Infrared spectra (FTIR) were recorded at KAIST energy and environment research center with a Jasco FT/IR-4100 type-A spectrometer using KBr pellets. Thermogravimetric analyses were performed on a Shimadzu DTG-60A by heating the samples up to 800 °C at a rate of 10 °C min^{-1} under nitrogen atmosphere. Elemental analysis was performed at the KAIST Central

Research Instrument Facility on a Thermo Scientific FLASH 2000 equipped with a TCD detector for carbon, nitrogen and hydrogen and on a Thermo Finnigan Flash EA 1112 for oxygen. Each experimental batch is measured twice and the elemental composition is given as a mean value of the two measurements. Textural characterization of polymers was carried out from nitrogen adsorption isotherms using a Micromeritics 3FLEX accelerated surface area and porosimetry analyzer at 77 K. Prior to measurement, samples were degassed at 423 K for 5 h under vacuum. The specific surface areas were derived from Brunauer–Emmett–Teller (BET) method. Pore size distribution was calculated with the Micromeritics 3FLEX software using a multi-walled carbon nanotube NLDFT model assuming cylindrical pore shape. This model was chosen as it gave the best fit between experiment and calculation. Q_{st} values were calculated with two temperatures using the 3Flex software. Moist CO_2 uptake was performed on a custom made TGA system. Moist gases are obtained by injecting the gas into a water bubbler (250 mL) before entering the TGA system. 6–8 mg of dry sample was degassed in dry N_2 (50 mL/min) at 120°C for one hour. After cooling down to 40 °C, the gas was changed to moist N_2 and mass uptake was measured for 2 h. As nitrogen uptake is several orders of magnitude lower than CO_2 in porous materials, it can be considered negligible and the collected data is used as background to determine the water uptake of the sample. After regeneration under vacuum at 100 °C overnight, the samples were degassed in dry N_2 (50 mL/min) at 120 °C for one hour. After cooling down to 40 °C, the gas was changed to moist CO_2 and mass uptake was measured for 2 h. The collected data from moist N_2 uptake is subtracted from the collected moist CO_2 data to give the actual moist CO_2 uptake reported in the main text.

Synthesis

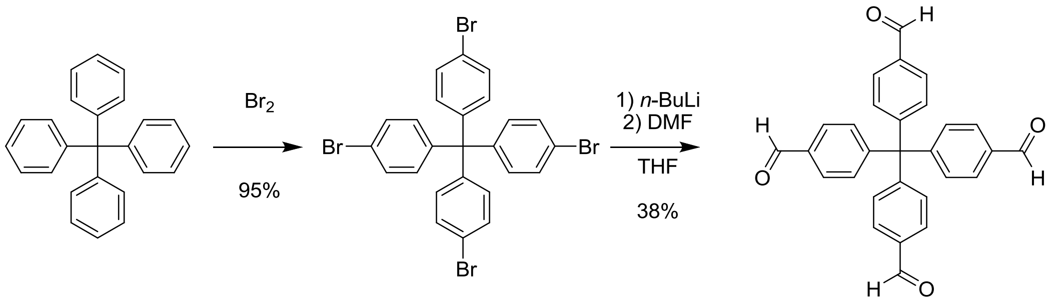

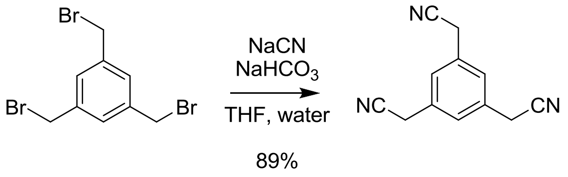

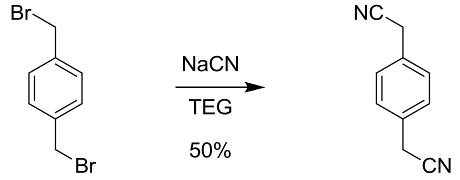

2,2'-(1,4-phenylene)diacetonitrile, 2,2',2''-(benzene-1,3,5-triyl)triacetonitrile and tetrakis(4-formylphenyl)methane were synthesized according to reported procedures.[S1-3]

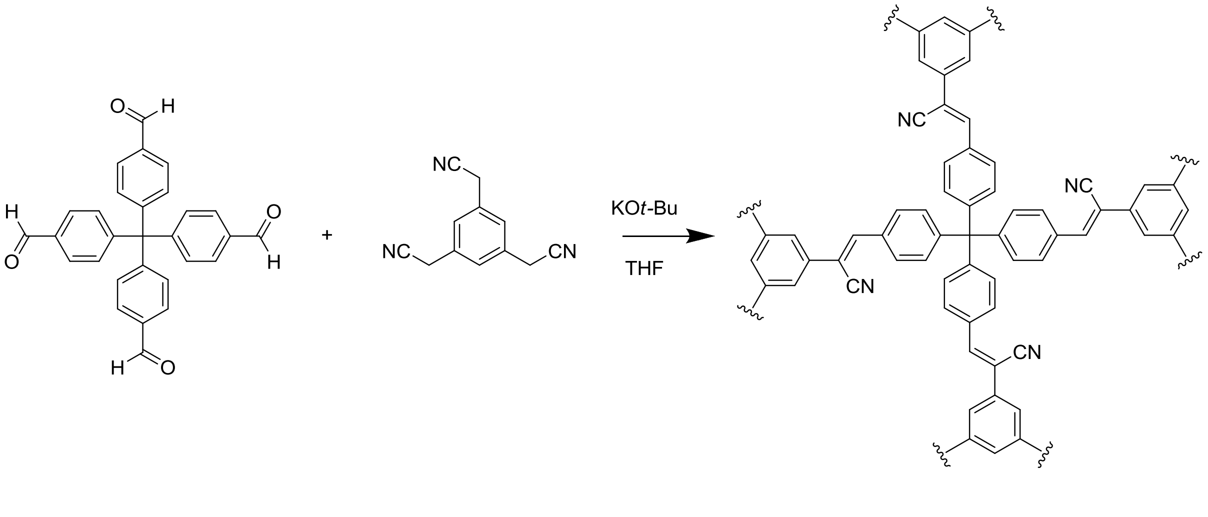

COP-156

Synthesis of COP−156 was performed in a similar manner as our previous work[S4] : 2,2',2''-(benzene-1,3,5-triyl)triacetonitrile (180 mg, 0.92 mmol) and tetrakis(4-formylphenyl)methane (300 mg, 0.69 mmol) were dissolved in dry THF (40 mL) at room temperature under nitrogen. Potassium *tert*-butoxide (311 mg, 2.78 mmol) in THF (40 mL) was added dropwise and the mixture was stirred for 12 hours at room temperature. Afterwards, the reaction mixture was poured into 200 mL of acidified ethanol (5% glacial AcOH). The resulting precipitate was separated by filtration, washed with ethanol and dried in vacuum at 80 °C overnight. COP−156 was afforded as a pale yellow solid (289 mg).

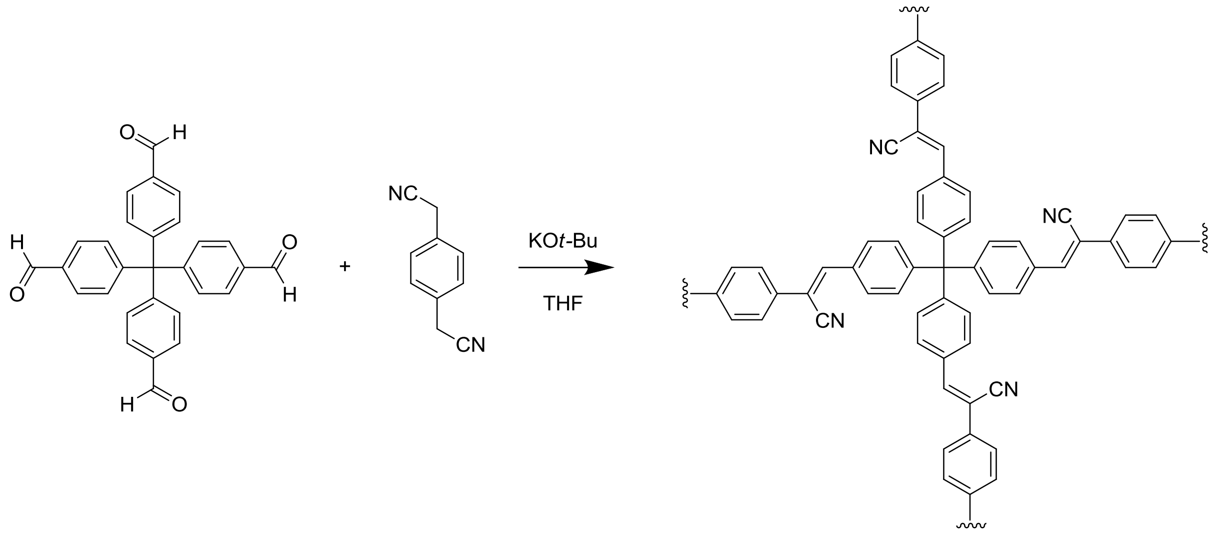

COP-157

Synthesis of COP−157: Same procedure as COP-156 using 2,2'-(1,4-phenylene)diacetonitrile (215 mg, 1.38 mmol). COP−157 was afforded as a pale yellow solid (232 mg).

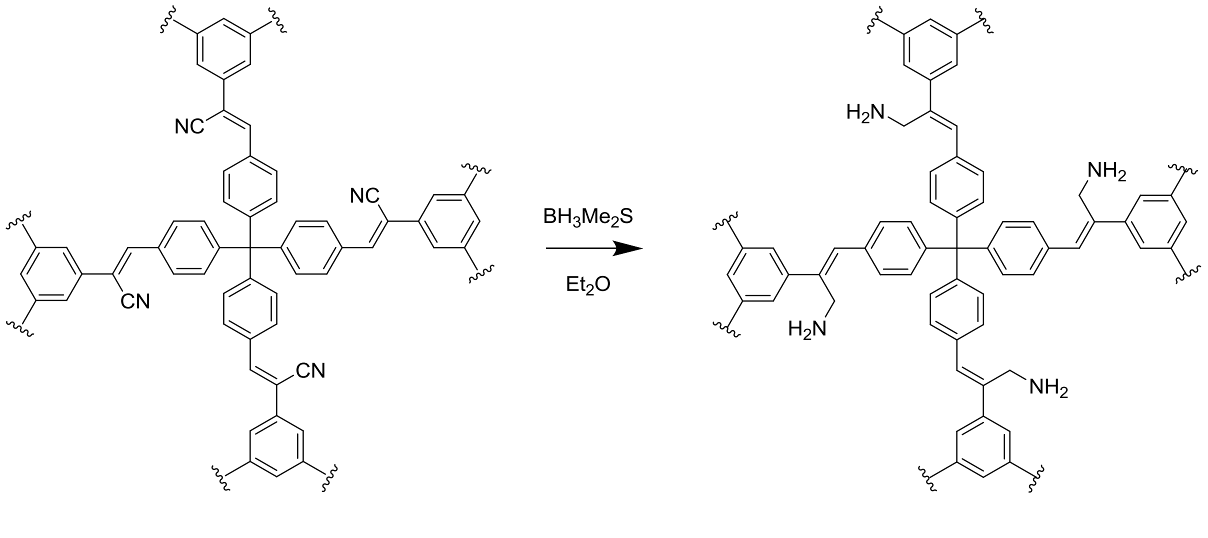

COP-156 **COP-156-AMINE**

Synthesis of COP-156-amine: COP-156 (150 mg) was dispersed in dry ether (5 mL) at room temperature under nitrogen. BH_3–Me_2S (5 M in ether, 2.5 mL) was added dropwise and the mixture was left to stir for 12 h. The mixture was quenched with MeOH, the solid filtered off and washed in a Soxhlet extractor for 24 h with MeOH. COP-156-Amine (127 mg) was afforded as an off-white powder after drying under vacuum at 80 °C for 12 h.

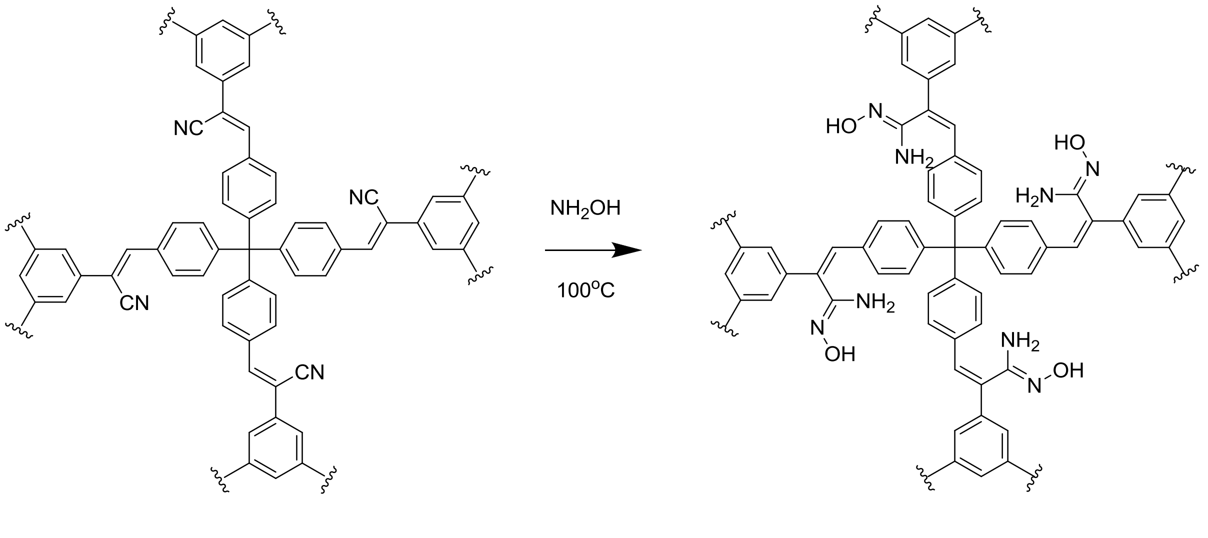

COP-156 **COP-156-AMIDOXIME**

Synthesis of COP-156-amidoxime: COP-156 (120 mg) was dispersed in an aqueous hydroxylamine solution (50 wt % in water, 10 mL) and heated to 100 °C for 12 h. The solid was filtered off and washed in a Soxhlet extractor for 24h with MeOH. COP-156-Amidoxime (92 mg) was afforded as a yellow powder after drying under vacuum at 80 °C for 12 h.

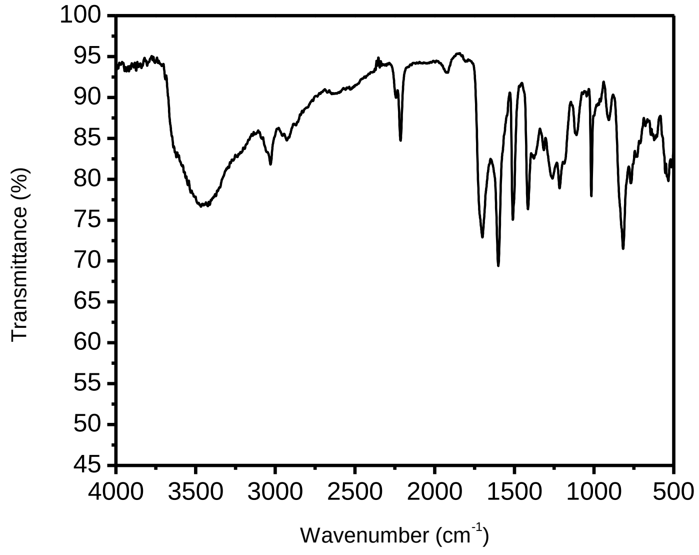

Figure S1: FTIR spectrum of COP-157.

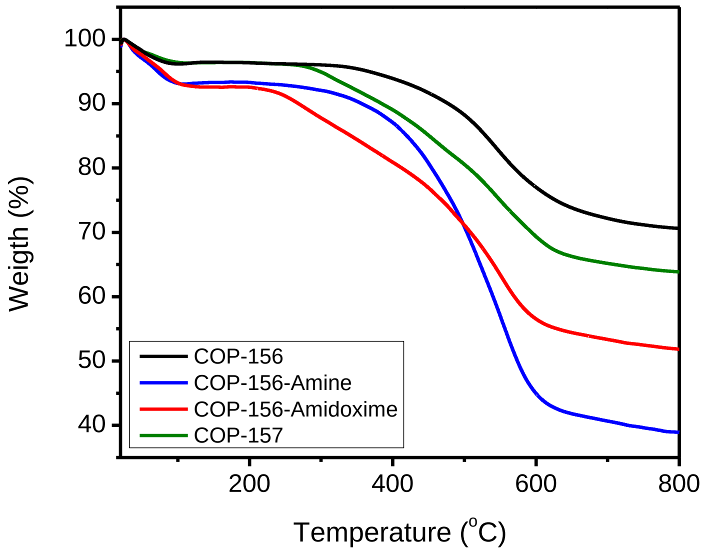

Figure S2: TGA analysis of COP-156 (black), COP-156-amine (blue), COP-156-amidoxime (red) and COP-157 (green).

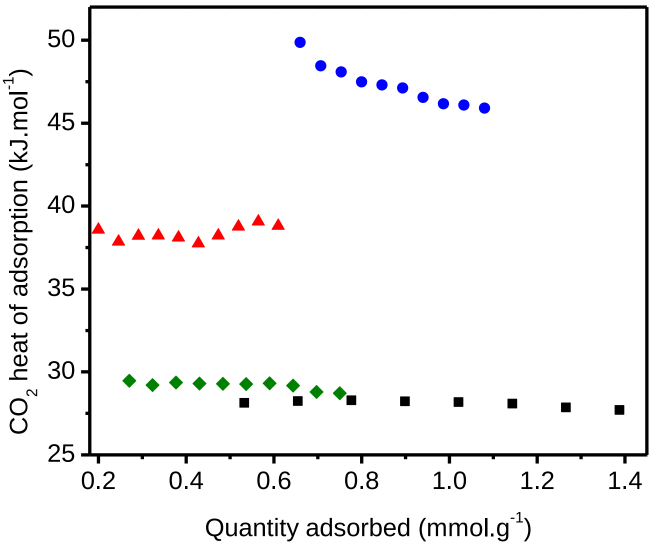

Figure S3: CO_2 heat of adsorption of COP-156 (black), COP-156-amine (blue), COP-156-amidoxime (red) and COP-157 (green).

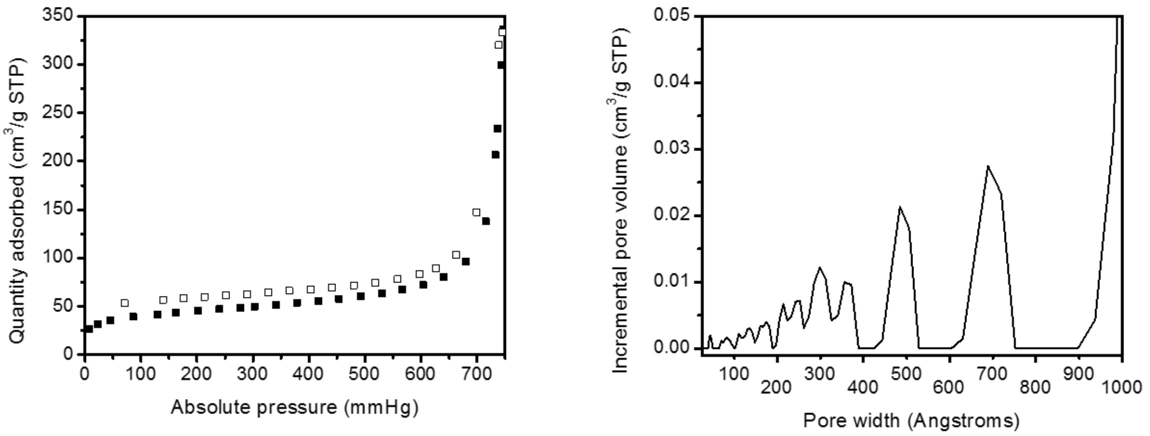

Figure S4: Left: Nitrogen adsorption (filled dots)-desorption (empty dots) isotherms at 77K; Right: Pore size distribution by NLDFT (N_2 77 K, cylindrical pores, multi-walled carbon nanotubes) of COP-157.

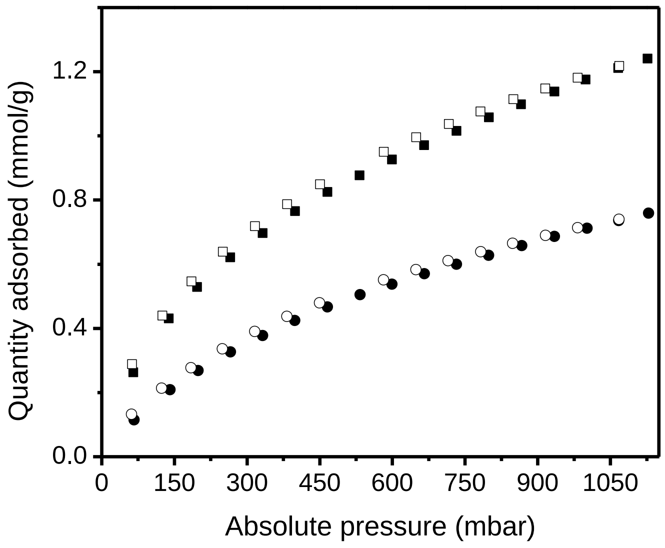

Figure S5: CO_2 adsorption (filled dots)-desorption (empty dots) isotherms at 273 K (square) and 298 K (circle) of COP-157.

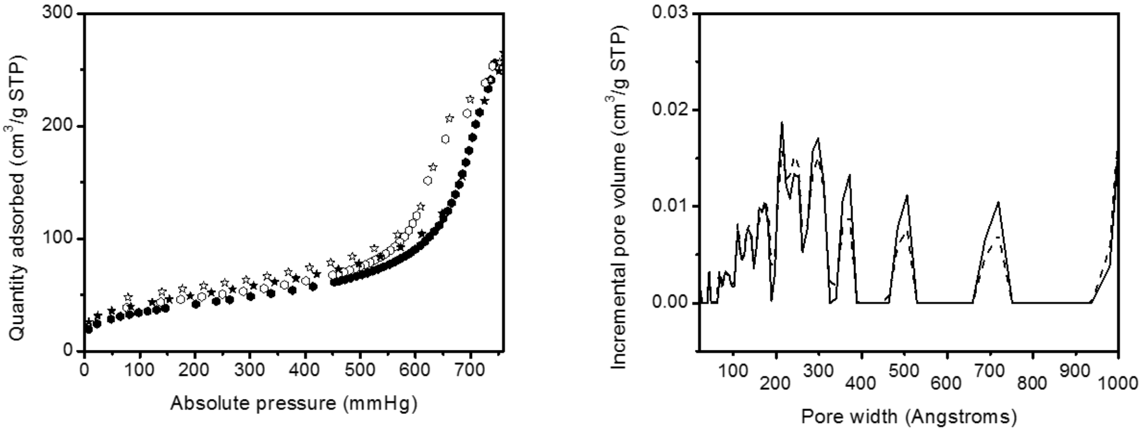

Figure S6: Left: Nitrogen adsorption (filled dots)-desorption (empty dots) isotherms at 77 K; Right: Pore size distribution by NLDFT (N_2 77 K, cylindrical pores, multi-walled carbon nanotubes) of COP-156-amine by $LiAlH_4$ (hexagon, solid line) and COP-156-amine by BH_3–THF (star, dashed line).

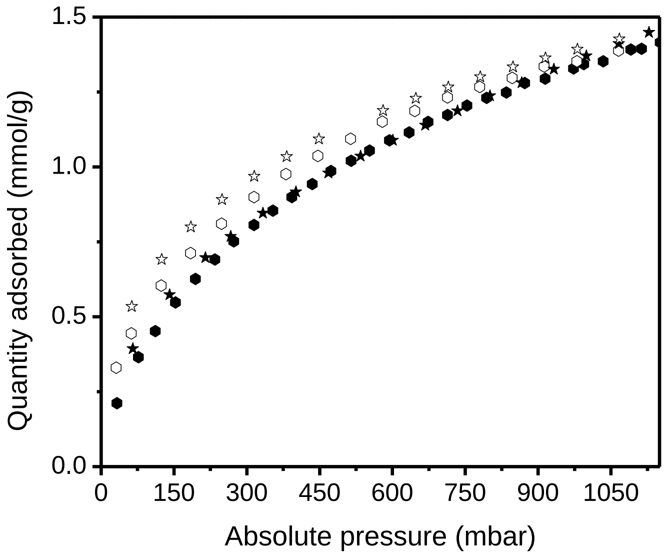

Figure S7: CO_2 adsorption (filled dots)-desorption (empty dots) isotherms at 273 K of COP-156-amine by $LiAlH_4$ (hexagon) and COP-156-amine by BH_3–THF (star).

Table S1: BET surface area, CO2 sorption properties at 273K of COP-156-Amine by different reducing agents.

**Structure**	**BET surface area (m^2/g)**	**CO_2 at 0 °C (mmol/g) 0.15/1bar**
COP-156-Amine ($LiAlH_4$)	141	0.58/1.36
COP-156-Amine(BH_3-THF)	165	0.55/1.35

Table S2: Elemental analysis of all networks

**Structure**	**C**	**H**	**N**	**O**
COP-156	79.73	4.21	7.86	4.13
COP-157	78.84	4.45	7.32	5.32
COP-156-amine (LiAlH4)	73.62	5.59	5.82	5.86
COP-156-amine (BH_3-THF)	70.49	5.55	4.55	9.15
COP-156-amine (BH_3-Me_2S)	69.90	5.30	5.30	9.44
COP-156-amidoxime	66.21	4.94	8.13	13.04

References

S1. Langer, P.; Anders, Joachim T. *Eur. J. Org. Chem.* **2002**, *2002*, 686.

S2. Aakeroy, C. B.; Smith, M. M.; Desper, J. *Cryst. Eng. Commun.* **2012**, *14*, 71.

S3. Fournier, J.-H.; Wang, X.; Wuest, J. D. *Can. J. Chem.* **2003**, *81*, 376.

S4. Ozdemir, E.; Thirion, D.; Yavuz, C. T. *RSC Adv.* **2015**, *5*, 69010.
